# Unraveling the relative abundance of psychobiotic bacteria in children with Autism Spectrum Disorder

**DOI:** 10.1038/s41598-024-72962-3

**Published:** 2024-10-17

**Authors:** Mennat-Allah K. Darwesh, Wafaa Bakr, Tarek E. I. Omar, Mohammed A. El-Kholy, Nashwa Fawzy Azzam

**Affiliations:** 1 Department of Microbiology. High Institute of Public Health, Alexandria University, Alexandria, Egypt; 2 Department of Pediatrics, Faculty of Medicine, Alexandria University, Alexandria, Egypt; 3 Department of Microbiology and Biotechnology, Division of Clinical and Biological Sciences, College of Pharmacy, Arab Academy for Science, Technology and Maritime Transport (AASTMT), Alexandria, Egypt

**Keywords:** Autism Spectrum Disorder, Dysbiosis, Gut-brain axis, Behavioural effect, Psychobiotics, Children, Microbiology, Molecular biology, Neuroscience, Psychology, Health care, Medical research

## Abstract

Autism Spectrum Disorder (ASD) is a complex neurodevelopmental disorder characterized by social deficits. Accumulated evidence has shown a link between alterations in the composition of gut microbiota and both neurobehavioural and gastrointestinal symptoms in children with ASD which are related to the genera *Lactobacillus and Bifidobacterium*. These genera have been recently categorized as “psychobiotics”. Moreover, this study aimed to compare the relative abundance of psychobiotics (*L. plantarum*, *L. reuteri*, and *B. longum*) to the total gut microbiome in typically developing (TD) children and those with ASD in order to correlate the distribution of psychobiotic with the severity and sensory impairments in autism. The ASD children were assessed using the Childhood Autism Rating Scale (CARS), while sensory impairments were evaluated using the Short Sensory Profile (SSP). Furthermore, the gut microbiome was analyzed using the quantitative real-time PCR. The study revealed a statistically significant increase in the relative abundance of *L. reuteri* and *L. plantarum* in the TD group in comparison to ASD children. Regarding the SSP total score of ASD children, a statistically significant negative correlation was found between both *Lactobacillus* and *L. plantarum* with the under-responsive subscale. For the Autism Treatment Evaluation Checklist (ATEC) score, *B. longum* and *Lactobacillus* showed a significant positive correlation with Health/Physical/Behaviour.

**Supplementary Information:**

The online version contains supplementary material available at 10.1038/s41598-024-72962-3.

## Introduction

Autism Spectrum Disorder (ASD) is described as the presence of restricted, repetitive patterns of behaviours, activities, interests or/ and impairments in social communication. It is a lifelong neurodevelopmental disorder that appears during infancy and early childhood^1^. In Egypt, the estimated prevalence of ASD was 5.4 per 1000 children^2^.

The etiopathogenesis of ASD is multifactorial, which results from a complex interplay between genetic and environmental factors^3^. Moreover, gut dysbiosis is a specific environmental factor that triggers the development of ASD and commences emotional and behavioural attributes, thus reflecting a strong gut-brain axis link^4–6^. Since there is no definite distinctive profile of altered microbial composition in ASD, investigating the gut microbial diversity of patients can help to identify certain microbial biomarkers for ASD^7^.

Interestingly, certain beneficial bacterial genera (*Lactobacillus* and *Bifidobacterium*), which are commonly-used probiotics and recently named as “psychobiotic”, these psychobiotics are live organisms that provide health benefits to individuals suffering from mental illnesses when ingested in appropriate quantities. These bacteria belong to a type of probiotics capable of producing and delivering neuroactive substances that act on the brain- gut axis. Moreover, psychobiotics have antidepressant and anxiolytic effects, characterized by changes in neurological, cognitive, emotional, and systemic indices. They achieve these effects through the modulation of neural networks linked to emotional attention, exerting their positive effects on brain function^8,9^. Furthermore, they have reduced the severity of ASD symptoms via their effect on re-establishing the healthy balance of gastrointestinal (GI) microbiota, and modulated the levels of tissue neurotransmitters. Both genera are able to produce GABA, which is the main inhibitory neurotransmitter in the brain as lower levels of GABA are often associated with both anxiety as well as social disorders in ASD individuals^10^. In addition, some psychobiotic bacteria protect against the overgrowth of pathogens by maintaining the epithelial barrier integrity^11^. They also play a role in the metabolism of some dietary compounds, toxins, and drugs^7^. Many studies have shown differences in the composition of these bacterial genera in the gut microbiome of ASD children in comparison to typically developing (TD) children^4,6,7^.

Other species, such as *Lactobacillus gasseri* CP2305, *Lactobacillus casei* Shirota, *and Bifidobacterium breve* CCFM1025, can be used as psychobiotics and have been associated with improvements in alleviating symptoms of depression and anxiety, reducing stress-associated behaviors, decreasing cognitive and somatic anxiety scores, enhancing sleep quality, and lowering perceived stress levels^12–14^.

Two *Lactobacillus* species, *Lactobacillus plantarum* (*L. plantarum*) and *Lactobacillus reuteri* (*L. reuteri*), proved to have mechanistic insight into the gut-brain-axis signaling, which influence the functioning of the central nervous system, and mood through the production of neuroactive metabolites^15,16^. Moreover, *L. plantarum* can improve certain ASD symptoms particularly those linked with rule-breaking and disruptive behaviours. A study in Taiwan investigated the effects of the administration of *L. plantarum* PS128 probiotics on boys with ASD, and reported significant amelioration of opposition/defiance behaviours, anxiety, irritability, hyperactivity, rule-breaking, communication behaviour, and the total assessment score of questionnaires used in research and clinical context to assess numerous psychological and behavioral outcomes in ASD children^17^. As regards *L. reuteri*, it improved social behaviour in ASD patients due to the increased levels of oxytocin (love hormone)^18^ as it can restore ASD-like phenotypes and dysbiosis when induced in mice^19^.

*Bifidobacterium* genus has strong anti-inflammatory properties and immunomodulatory activities through enhancing IL-10 or IL-12 synthesis by dendritic cells^20^. Furthermore, *Bifidobacterium longum* (*B. longum*) could palliate autistic-like behaviours (learning and memory ability, repetitive stereotyped behaviour, and despair mood) in addition to regulating the kynurenine pathway metabolism centrally (brain) and in the periphery system (gut and blood). It significantly regulates the quinolinic acid, and glutamic acid levels in the brain, and attenuates microglia activity in the cerebellum^21^. The probiotic strain *B. longum* 1714 has also shown an anti-stress effect and enhancement of the neurocognitive function in healthy mice^22^.

Probiotic-based therapy has been suggested as a potential low-risk complementary supplement that can improve behavioural symptoms and reduce GI tract-related complaints in children with autism^23^. Nonetheless, the selection of a probiotic is often made without prior investigation^24,25^. As many bacterial strains in probiotics already exist in the colon, detecting and characterizing their relative abundance in the gut of ASD children before prescription would significantly contribute to achieving a more specific and personalized choice of an optimal probiotic regimen for therapeutic intervention.

To the best of our knowledge, only few data is available on the relative abundance of psychobiotic bacteria in ASD and its correlation with the severity of ASD^26,27^. Hence, the aim of the current work was to detect the relative abundance of psychobiotic (*Lactobacillus*,* L. plantarum*, *L. reuteri*,* Bifidobacterium* and *B. longum*) in TD children as well as children with ASD in addition to assessing its correlation with the severity of ASD to ensure the use of probiotics in ASD.

## Methods

### Participants

A total of 87 ASD children only were included in the present study. They were admitted and assessed by the Autism Clinic of Alexandria University Children’s Hospital. However, ASD children with known syndromes, inflammatory bowel disease, diabetes mellitus, hepatic disease, known food intolerances or immunodeficiency, or those taking antibiotics and /or probiotics in the last three months were excluded from the study. A group of 36 cross-matched unrelated normal TD children of the same sex and age was also included as a reference TD group for the Egyptian gut microbiome profile. Prior to participation, a written informed consent was obtained from each participant’s parent or legal guardian. The consent process involved providing detailed information about the study’s purpose, procedures, potential risks, and benefits. The parents or legal guardians were given the opportunity to ask questions and were assured that their child’s participation was voluntary and could be withdrawn at any time without any consequences.

### Case severity and sensory impairment assessment

Detailed medical history and complete physical examinations were conducted for all children in the study, with particular emphasis on neurological examinations. Moreover, these ASD children were assessed according to the Diagnostic and Statistical Manual of Mental Disorders Fifth Edition [DSM-5] criteria^28^, while the severity of autism was evaluated based on the Childhood Autism Rating Scale [CARS]^29^.

The Autism Treatment Evaluation Checklist (ATEC) and the caregiver questionnaire were both used for the assessment of autistic behaviour and functioning in ASD cases. The ATEC consists of four subscales; sociability [20 items; with score range 0–40], speech/language communication [14 items; with score range 0–28], Health/Physical/Behaviour [25 items; with score range 0–75] and sensory/cognitive awareness [18 items; with score range 0–36]. The ratings on subscales I–III range from 0 to 2, where a score of 0 indicated higher developmental ability and lower severity of autistic and behavioural problems. The total ATEC score was calculated by summing up the score of the respective subscale. The items related to the Health/Physical/Behaviour were scored from 0 [‘no problem’] to 3 [‘serious problem’]. The maximum score on this scale is 179 [range 0–179]. A reduction in score indicated improvement, while higher scores indicated more difficulties^30^.

The presence or absence of sensory impairment was evaluated using a short sensory profile [SSP]^31^. The SSP comprises 38 items that are rated by caregivers, with a scale from 1 to 5 points for each item. Additionally, the SSP seven sections included taste/smell sensitivity, tactile sensitivity, movement sensitivity, auditory filtering, under-responsive/seek sensation, visual/auditory sensitivity, and low energy/weak. The total score of the cases was calculated and then categorized either as having typical performance, definite sensory impairment, or probable sensory impairment. Both category scores and the total score were then interpreted according to the SSP and considered as independent variables, with the total score being identified as the most accurate indicator of the sensory impairment.

The modified short version of the GI Severity Index, specifically the 6-GSI questionnaire, was used to evaluate gastrointestinal (GI) symptoms^32^. It consists of 6 items (diarrhea, constipation, stool smell, stool consistency, abdominal pain, and flatulence). Based on its frequency per week, each symptom scored either 0, 1, or 2; where 0 means the absence of the symptom, while scores 1 and 2 denote the presence of the symptom with different severity. A total score of 3 or lower was categorized as low, while a score higher than 3 was considered high.

### Gut microbiome analysis

#### Sample collection, preservation, and transportation

Stool samples were obtained from both ASD and TD children, preserved in the freezer immediately after defecation at home, transported frozen to the Gut Microbiome Laboratory at Alexandria University, and stored at -80 °C until further processing.

### Bacterial DNA extraction

DNA was extracted from each stool sample (180 mg) using QIAamp DNA Stool Extraction Mini Kit (Qiagen, Germany). The extracted DNAs were kept at -80 °C; their amplification and analysis were performed using the quantitative real-time polymerase chain reaction technique (qPCR).

### Relative abundance of psychobiotics using quantitative real-time PCR (qPCR)

Initially, universal primer pairs, targeting a conserved region of the 16S rDNA sequence found in all bacteria, were selected to amplify the total bacterial population present in the samples. Afterwards, specific primer pairs, targeting unique regions of the 16S rDNA sequence of each bacterial species, were chosen for the amplification of the genus *Lactobacillus*, including *L. plantarum* and *L. reuteri*, as well as the *Bifidobacterium* genus, including *B. longum*. All primers (Invitrogen, USA) are listed in (Supplementary Table S1)^33–35^.

The amplification was performed using the SensiFAST TM SYBR No-ROX PCR kit (Bioline Co. USA) in a light cycler (Rotor Gene Q, Qiagen, Germany). The thermal cycling conditions included initial denaturation for 10 min at 95 ˚C, followed by 40 cycles of denaturation for 30 s at 95 ˚C, annealing for 30 s at 60 ˚C, and extension for 30 s at 72 ˚C. A melting curve analysis was conducted to check the specificity of the amplified products and the relative quantitation was automatically calculated^35^.

During the qPCR, the universal primer pair, which served as a denominator for comparison, amplified the conserved region of the 16S rDNA sequence, while the specific primer amplified the target regions of interest. The relative abundance of each bacterial species was automatically determined relative to the total bacterial count by the Rotor-Gene software and expressed as a relative fold difference.

### Statistical analysis

The obtained data were statistically analysed using IBM SPSS software package version 20.0 (IBM Corp, Armonk, NY). The Categorical data were presented in numbers and percentages, with the Chi-square test employed to compare the two groups. The Fisher Exact correction test was conducted in cases where more than 20% of the cells had an expected count of less than 5. Continuous data were assessed for normality using the Shapiro-Wilk test. For normally distributed quantitative variables, the data were expressed as a range (minimum and maximum), mean, standard deviation, and median. For non-normally distributed quantitative variables, the Mann-Whitney test compared two groups, while the Kruskal-Wallis test compared different groups. Pairwise comparisons were conducted using the Post Hoc test (specifically Dunn’s test for multiple comparisons). Statistical significance was considered at the 5% level.

## Results

### Demographic and clinical data of the participants

The demographic data revealed that out of the 87 ASD children, 58 (66.7%) were males and 29 (33.3%) were females, with a male to female ratio of 2.8:1. Children’s age ranged from 3 to 15 years old with a mean age of 6.97 ± 2.98 years. In the TD group, 20 subjects (55.6%) were males, and 16 subjects (44.4%) were females with a male to female ratio of 1.5:1. Their ages ranged from 2 to 12 years with a mean age of 5.79 ± 2.71 years.

Concerning the relative abundance of psychobiotic bacteria and sex difference, the *Bifidobacterium* genus was significantly reduced in females (2.91E-2) in comparison to males (1.25E-1) in TD group (*p* = 0.015) (Supplementary Table S2). However, these differences among ASD children were not statistically significant (Supplementary Table S3).

According to CARS, 66 (75.9%) out of the 87 ASD children had mild to moderate ASD (CARS < 36), while only 21 (24.1%) had severe ASD (CARS ≥ 36) with a mean score of 33.64 ± 5.14 (Supplementary Table 4). For the ATEC, the mean of the total score was 79.63 ± 24.88 and ranged from 41 to 118 (Supplementary Table 4). A descriptive analysis of ASD children according to the ATEC subscale is shown in (Supplementary Table S5).

Regarding the SSP total score, out of 87 ASD children, 73 (83.9%) had a definite sensory impairment, 12 (13.8%) had a probable impairment and only 2 (2.3%) had a typical performance. The mean SSP score was 127.94 ± 16.07. The highest definite impairment was under responsiveness and seeking sensation (90.8%), followed by tactile sensitivity and auditory filtering (67.8% and 65.5%, respectively), while the lowest definite impairments were in movement and visual sensitivity (19.5 and 18.4%, respectively) (Supplementary Table S6).

Concerning the GI symptoms, out of the 87 ASD children, 77 (88.5%) had at least one GI symptom, while 10 (11.5%) had no symptoms. The mean 6-GSI score was 3.37 ± 2.23; 35 (40.2%) had a moderate score (≤ 3), while 42 (48.3%) had a severe score (> 3) (Supplementary Table S7). The most prevalent GI symptoms reported in ASD children were unusual stool smell (41%), followed by flatulence and constipation (26.4% each), watery stool consistency (4.7%), abdominal pain (4.6%), and diarrhoea (3.4%). None of the TD group had GI symptoms.

### Psychobiotics analysis

#### *Lactobacillus*, *L. plantarum* and *L. reuteri*

**Fig. 1:**
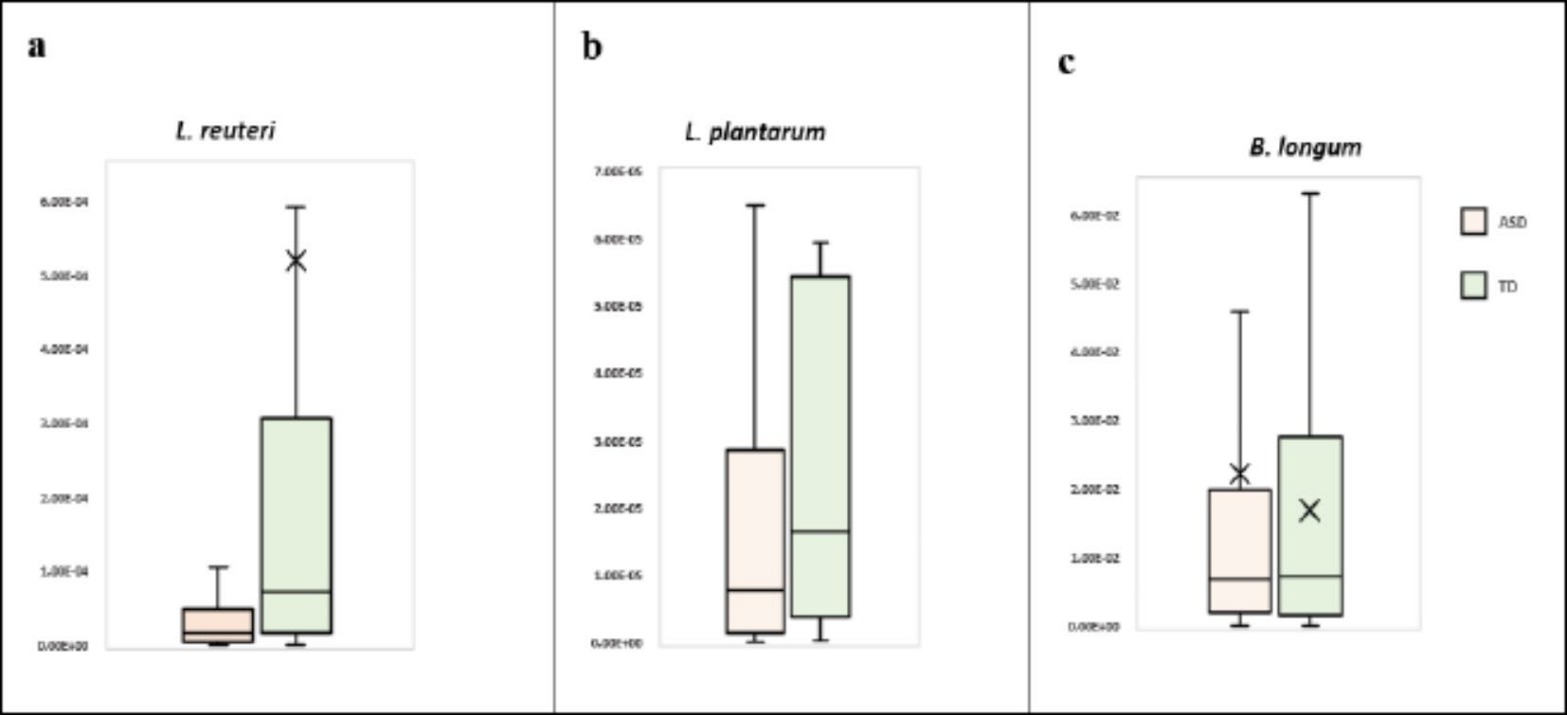
Box and whisker graph of the relative abundance of *L. reutri*, *L. plantarum*, and *B. longum* in the studied groups, the thick line in the middle of the box represents the median, the box represents the inter-quartile range (from 25th to 75th percentiles), the whiskers represents the minimum and maximum.

*Lactobacillus* genus did not differ significantly among ASD children and the TD group (3.66E-03 versus 3.21E-03, respectively, *p* = 0.332). Nonetheless, the two *Lactobacillus* species were significantly reduced in the ASD group in comparison to the TD group. The relative abundance of *L. reuteri* was 7.26E-5 in the TD group versus 1.59E-5 in ASD cases (*p* = 0.005) and that of *L. plantarum* was 1.64E-5 in the TD group versus 7.83E-6 in ASD cases (*p* = 0.039) (Table 1; Fig. 1A and B).

**Table 1:** Comparison between the two studied groups according to the psychobiotic bacteria.

Bacteria			U	*p*-value
	ASD group (*n* = 87) Median (IQR)	TD group (*n* = 36) Median (IQR)		
*Lactobacilli*	3.66E-3 (1.38E-2)	3.21E-3 (3.71E-2)	1391.5	0.332
*L. reuteri*	1.59E-5 (4.79E-5)	7.26E-5 (3.0E-4)	1061.0	0.005*
*L. plantarum*	7.83E-6 (2.85E-5)	1.64E-5 (5.26E-5)	1194.5	0.039*
*Bifidobacterium*	5.09E-2 (1.23E-1)	5.32E-2 (1.86E-1)	1382.0	0.306
*B. longum*	6.49E-3 (1.72E-2)	7.31E-3 (2.74E-2)	1547.5	0.918

IQR: Inter quartile range.

U: Mann Whitney test.

*p*-value: *p* value for comparing between the two studied groups.

*: Statistically significant at *p* ≤ 0.05.

#### *Bifidobacterium* and *B. longum*

Children with ASD showed no significant difference in the relative abundance of *Bifidobacterium* (5.09E-02) versus the TD group (5.32E-02) (*p* = 0.306). Also, there was no statistical difference between the two groups regarding *B. longum* (6.49E-3 versus 7.31E-3 in ASD and TD groups, respectively) (*p* = 0.918) (Table 1; Fig. 1C).

### Psychobiotics and autism severity

According to CARS score, it was found that *Bifidobacterium*,* L. plantarum*, and *L. reuteri* were higher in mild to moderate ASD children, while *Lactobacillus* genus and *B. longum* were higher in severe ASD children. Nevertheless, none of these differences were statistically significant. Statistically, there was not any significant correlation between any of the bacteria understudy and the CARS score (Table 2).

**Table 2:** Association between autism severity and psychobiotic bacteria in ASD children.

Bacteria	Mild to moderate (*n* = 66)	Severe (*n* = 21)	Mann-Whitney U	*p*-value	Spearman *r*
*Lactobacillus*	3.51E-3–1.60E-2	3.66E-3–1.02E-2	651	0.677	-0.034
*L. reuteri*	1.85E-5–3.88E-5	1.49E-5–5.90E-5	642	0.613	0.161
*L. plantarum*	8.71E-6–2.76E-5	3.76E-6–1.65E-5	610	0.410	-0.181
*Bifidobacterium*	5.52E-2–1.03E-1	4.49E-2–1.19E-1	626	0.506	-0.031
*B. longum*	5.65E-3–1.84E-2	9.41E-3–1.36E-2	605.5	0.385	0.165

Variable statistics represented by Median and IQR.

IQR: Inter quartile range.

U: Mann Whitney test.

*p*-value: *p* value for comparing between Mild to Moderate (≤ 35) and Severe (> 35).

r_s_: Spearman coefficient.

As for the ATEC score, using Spearman’s correlation (r_s_), *B. longum* showed a significant positive correlation with health/ physical/ behaviour and sociability subscales (r_s_=0.344, *p* = 0.043, and r_s_=0.335, *p* = 0.049, respectively). *Lactobacillus* also showed a significant positive correlation with the health/ physical/ behaviour subscale (r_s_=0.360, *p* = 0.034). On the other hand, *Bifidobacterium*, and *L. plantarum* showed a significant negative correlation with the sensory/ cognitive awareness subscale (r_s_=-0.381, *p* = 0.024, and r_s_=-0.336, *p* = 0.048, respectively) (Table 3).

**Table 3:** The correlation between ATEC subscales and the psychobiotic bacteria understudy in ASD group.

Variables	*Lactobacillus*		*L. reuteri*		*L. plantarum*		*Bifidobacterium*		*B. longum*	
	r_s_	*p*	r_s_	*p*	r_s_	*p*	r_s_	*p*	r_s_	*p*
Speech/Language/ Communication	-0.008	0.962	-0.095	0.589	-0.167	0.338	-0.275	0.110	0.003	0.987
Sociability	0.180	0.301	-0.055	0.754	-0.004	0.984	0.147	0.398	0.335	0.049*
Sensory/Cognitive awareness	0.014	0.938	-0.042	0.810	-0.336	0.048*	-0.381	0.024*	-0.196	0.260
Health/Physical/Behaviour	0.360	0.034*	0.025	0.885	0.010	0.955	0.106	0.544	0.344	0.043*
Total Score ATEC	0.177	0.308	-0.064	0.714	-0.164	0.347	-0.069	0.695	0.207	0.233

r_s_: Spearman coefficient.

*: Statistically significant at *p* ≤ 0.05.

**Fig. 2:**
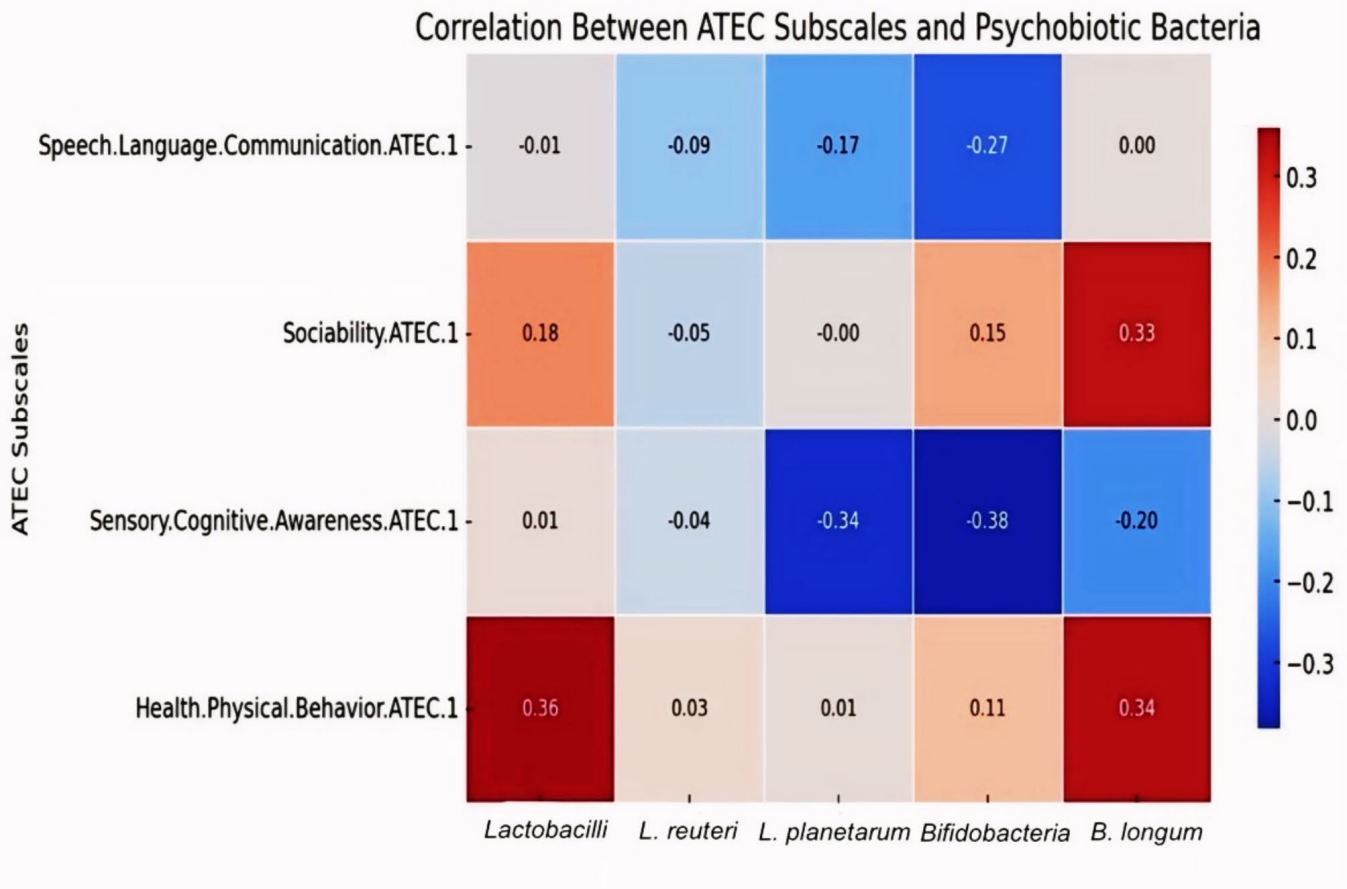
Heatmap of the correlation between ATEC subscales and psychobiotic bacteria in children with ASD. The heatmap was generated using the Python programming language (version 3.8.10) with the Seaborn (version 0.11.2) and Matplotlib (version 3.4.2) libraries. The color coding scale ranges from blue to red, indicating the strength and direction of each correlation, where blue indicates negative correlations and red indicates positive correlations.

Figure 2 illustrates the correlation between ATEC subscales and psychobiotic bacteria in ASD children. It shows the Spearman correlation coefficients, with color coding indicating the strength and direction of each correlation. The key findings include significant negative correlations of sensory/cognitive awareness with *L. plantarum* and *Bifidobacterium*, and positive correlations of sociability and Health/Physical/Behaviour with *B. longum*, highlighting potential gut-brain interactions in ASD.

### Psychobiotics and sensory impairment

As shown in Table 4, there was no statistically significant difference in the bacteria understudy between the ASD subgroups of definite impairment, probable sensory impairment and typical sensory performance.

**Table 4:** Relationship between SSP total score and psychobiotic bacteria in ASD group.

Bacteria	Definite Impairment Median (IQR)	Probable Impairment Median (IQR)	Typical Performance Median (IQR)	H	*p*-value
*Lactobacilli*	3.17E-3 (9.52E-3)	1.14E-2 (3.22E-2)	5.80E-4 (5.16E-4)	2.164	0.339
*L. reuteri*	1.50E-5 (4.56E-5)	1.94E-5 (2.53E-5)	3.08E-5 (3.67E-6)	0.117	0.943
*L. plantarum*	6.14E-6 (2.38E-5)	1.58E-5 (8.19E-5)	9.71E-5 (1.61E-4)	2.536	0.281
*Bifidobacterium*	5.09E-2 (1.02E-1)	4.35E-2 (1.32E-1)	4.25E-2 (6.22E-2)	0.779	0.677
*B. longum*	8.00E-3 (2.08E-2)	4.61E-3 (7.93E-3)	2.58E-3 (2.39E-3)	4.493	0.106

IQR: Inter quartile range.

H: H for Kruskal Wallis test.

*p*: *p* value for comparing between the three subgroups of SSP Total Score.

Similarly, there was not any significant correlation between the bacteria understudy and the SSP total score. Nonetheless, there was a statistically significant negative correlation between the *Lactobacillus* and *L. plantarum* with under-responsive subscale, in which the higher the bacteria, the more severe is the impairment (*p* = 0.027 and 0.038, respectively) (Table 5, Supplementary Figure S1, S2).

**Table 5:** The correlation between SSP score and psychobiotic bacteria in ASD children.

Variables	*Lactobacillus*		*L. reuteri*		*L. plantarum*		*Bifidobacterium*		*B. longum*	
	r_s_	*p*	r_s_	*p*	r_s_	*p*	r_s_	*p*	r_s_	*p*
Tactile	-0.059	0.587	0.083	0.444	0.157	0.146	-0.096	0.376	-0.125	0.250
Taste/smell	0.193	0.073	-0.018	0.866	0.135	0.214	0.002	0.984	-0.049	0.650
Movement	-0.036	0.739	0.027	0.803	0.057	0.598	0.086	0.430	0.037	0.733
Under responsive	-0.237	0.027*	-0.098	0.366	-0.223	0.038*	-0.098	0.365	-0.009	0.933
Auditory	-0.063	0.561	-0.143	0.187	0.020	0.853	0.092	0.399	-0.035	0.744
Low Energy	0.184	0.088	0.057	0.603	0.001	0.994	0.048	0.657	-0.008	0.942
Visual	0.058	0.593	0.091	0.403	-0.013	0.905	0.153	0.157	0.102	0.346
SSP Total Score	0.037	0.733	-0.022	0.842	0.039	0.723	0.016	0.880	-0.023	0.830

r_s_: Spearman coefficient.

*: Statistically significant at *p* ≤ 0.05.

**Fig. 3:**
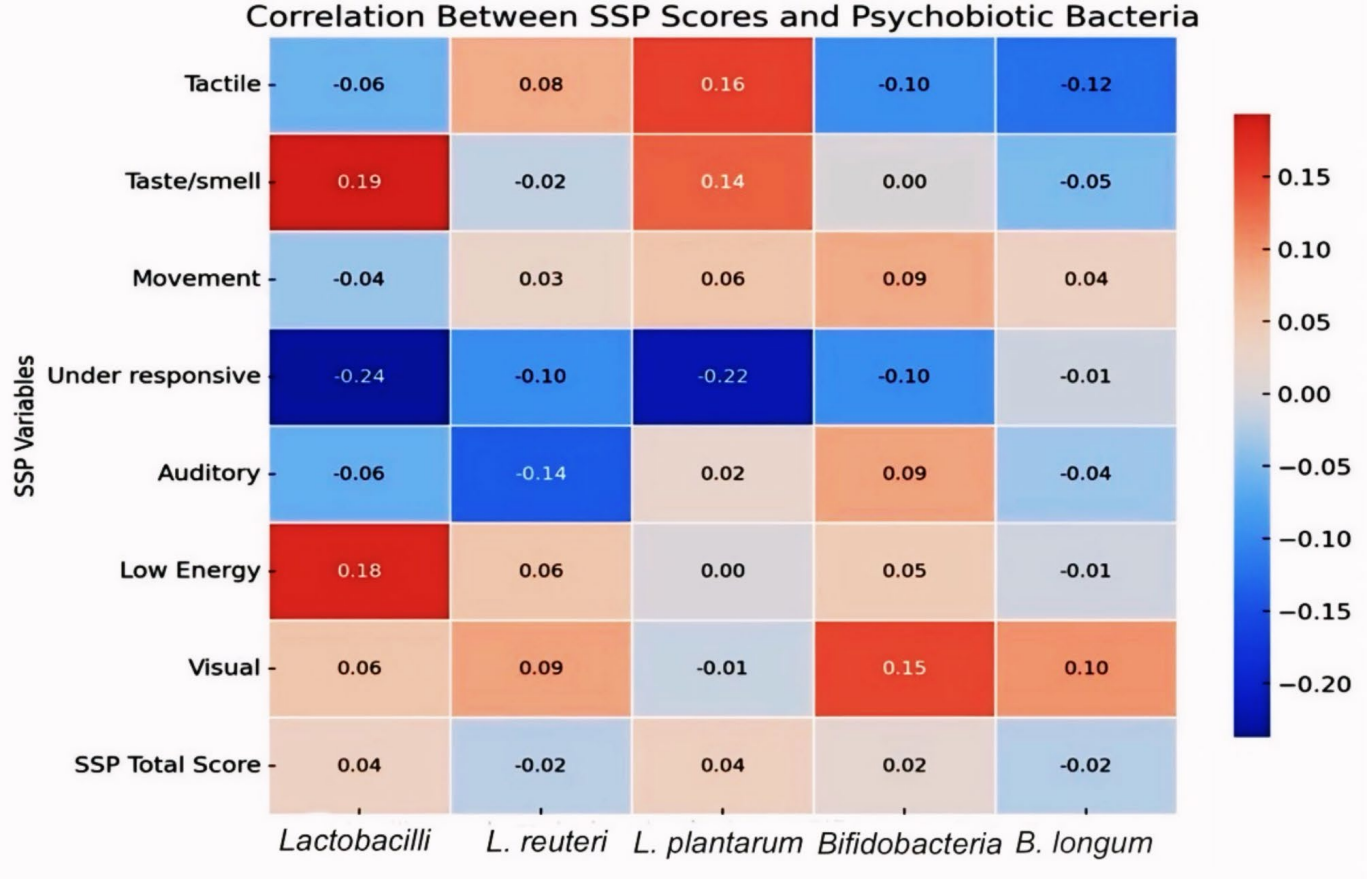
Heatmap of the correlations between psychobiotic bacteria and short sensory profile scores in children with ASD. For details on software and color-coding, refer to Fig. 2.

Figure 3 displays the Spearman correlation coefficients between various SSP scores and levels of specific psychobiotic bacteria in children with ASD. The color scale ranges from blue to red, where blue indicates negative correlations and red represents positive correlations.

Notably, under-responsive behaviours show significant negative correlations with both *L. plantarum* and *Bifidobacterium*, that is when sensory under-responsiveness increases, the level of these bacteria decreases. On the other hand, there were no significant positive correlations to be highlighted, but some non-significant positive trends were observed with *Lactobacillus* and *B. longum* in various SSP scores.

### Psychobiotics and the 6-GSI score

As demonstrated in supplementary Table S7, only *B. longum* differed significantly according to the 6-GSI score (*p* = 0.041), where it was the highest in children with moderate score. In addition, there was no significant correlation between the bacteria understudy and the GI severity subgroups (Supplementary Table S7). None of the GI symptoms differed significantly according to the relative abundance of different bacteria.

## Discussion

Different bacterial species within the same genus may have different functional roles and interactions with the host. In fact, analysis of the gut microbiome at the species level can provide deeper insights into the potential role of specific microbial species in the development and progression of ASD.

In the present study, the relative abundance of *Lactobacilli* and *Bifidobacteria* did not differ at the genus level, which was in accordance with the findings of Chamtouri et al.^27^, from Tunisia, and Tomova et al., from Slovakia^36^. In previous studies, *Lactobacillus* usually varied between TD and ASD children and a low level of *Bifidobacterium* served as a biomarker of ASD^4–7,37–41^. However, contradictory results between various studies can be partly attributed to the influence of genetics and epigenetics^42,43^, or some factors that may affect the microbiota such as diet, lifestyle, ethnicity, and environment^44–46^.

Although no variations were identified between the two groups understudy at the genus level, *L. reuteri* and *L. plantarum* species were significantly lower in the ASD group in comparison to the TD group. Chamtouri et al. compared *Lactobacillus* species profile in ASD and TD groups and reported the abundance of *L. plantarum* species in the TD group^27^. Parracho et al. focused on behavioural features, reporting improvement after *L. plantarum* WCSF1 administration in ASD children^47^.

Despite the high abundance of *Bifidobacterium*,* L. plantarum*, and *L. reuteri* in mild to moderate ASD cases in comparison to severe cases, there was no significant difference in the bacteria understudy in terms of autism severity according to CARS. The observed differences might be attributed to dietary preferences among ASD children. A similar finding was also reported by a previous Egyptian study^4^, and another study conducted by Laghi et al.^48^, who highlighted the inexistence of links between autism severity and the bacteria’s relative abundance. A similar observation of a reduced population of *Bifidobacterium* genus in severely autistic individuals was reported by Finegold et al.^49^. In addition, Chamtouri et al.^27^, in Tunisia reported a minor difference in *Lactobacillus* species diversity between children with severe and mild to moderate ASD. Regarding the potential contribution of the gut microbiome to autism severity, evidence may be inconclusive. Furthermore, clinical survey data indicated that children with ASD have a significantly higher risk of GI symptoms when compared to TD groups, where the severity of autism is associated with the incidence of GI symptoms^50,51^. To the best of the authors’ knowledge, most of the studies did not address potential confounding factors in a comprehensive way as they did not use consistent statistical analysis, and thus yielded variable results^52^.

Results of the current study may also support the findings of Santocchi et al., who administrated a probiotic formulation of 8 strains including *B. longum* and *L. plantarum* to ASD preschoolers and observed no significant variations in autism severity. This could be attributed to the complex and diverse heterogeneity of ASD aetiology in humans as well as the distinct unbalanced microbiota targets in each ASD case, which may lead to potentially different effects of probiotics interventions^53^. Nevertheless, several studies showed that probiotic treatments can alleviate ASD symptoms^54–56^. Most of the intervention research that administered a mixture of probiotics to ASD patients reported at least minute (although not always statistically significant) reductions in the severity of ASD after using these probiotic strains. They attributed their positive results to the concomitant effects of different probiotic strains on different pathways and the interactivity between their metabolites, which boost the neuromodulator response. However, most of these results were based on subjective parent interviews or questionnaire assessments. Moreover, they differed in the criteria for ASD diagnosis and examined probiotic effects without taking into consideration the presence/absence of GI symptoms, which may dramatically affect the outcomes of probiotic therapy^57^.

Specific strains of *Lactobacillus* or *Bifidobacterium* may differ in their impact on each subscale of ASD due to the individual’s existing gut microbiome composition. Therefore, one of the aims of the current study was to assess the differences in ASD assessment scales and the relative abundance of certain probiotic species to determine if the imbalances of these species could be the basis for the varied manifestation. Results of the current study revealed that despite the inexistence of significant correlation between SSP and ATEC total scores, and any of the bacteria understudy, there was a significant improvement of the under-responsive subscale with an increased count of *Lactobacillus* genus and *L. plantarum*. This finding was supported by Liu et al., and Kong et al. who demonstrated that taking *L. plantarum* PS128 for 28 weeks could reduce the scores of the social responsiveness scale (SRS). In other words, it helped in improving the anxiety, irritability, cognition, hyperactivity, communication and rule-breaking behaviour of ASD children^17,58^.

Regarding the ATEC subscale, the increase in the *Lactobacillus* genus and *B. longum* was significantly correlated with the improvement of the health/ physical/ behaviour and sociability subscales. Meanwhile, an increase in the counts of *L. plantarum* and *Bifidobacterium* was associated with impairment of sensory/ cognitive awareness subscale. Wang et al. reported a reduction in ATEC total score over a two-month period, particularly in the scores of social interaction/speech/language and communication^59^. The specificity of effects among probiotic species was reported in previous studies, where the administration of *L. reuteri*, and not any other species of probiotics, could reverse social behavioural abnormalities rather than repetitive behaviours and anxiety^19,26^. In addition, administration of *L. plantarum* WCFS1 improved only disruptive antisocial behaviours, anxiety, and communication problems^60^. El Alfy et al. from Egypt reported that the administration of a probiotic composed of two *Lactobacillus* species did not affect sensory/ cognitive awareness^61^. A meta-analysis including 10 studies showed that probiotic supplementation (where the main constituents are *L. plantarum*, *L. infantis* and *B. longum*) did not improve the associated behavioural symptoms in children with ASD^24^. Niu et al. from China observed a decrease in ATEC scores among ASD children who received a combination of a training program with probiotic treatment than those with training only^62^. These findings may support the individualized approach to determine how specific probiotic strains could be beneficial. Moreover, species-level analysis of an individual’s gut microbiome profile and clinical symptoms should be assessed before blind administration of probiotics to ASD cases since most probiotics in the market contain a blended mixture of different *Lactobacillus* and *Bifidobacterium* strains.

In the current study, none of the GI symptoms showed significant variations because of the relative abundance of different bacteria understudy. Nonetheless, contradictory results were presented by Shaaban et al. and West et al.^24,63^, who reported a significant improvement in the total 6-GSI score with a significant reduction in the score of constipation, stool consistency, flatulence, and abdominal pain after probiotic supplementation. In the first report of probiotic treatment for Chinese ASD children, a decrease in GI score was observed after 4 weeks of treatment^62^. Also, Chen et al. reported that altered microbiota was associated with behavioural phenotypes rather than GI symptoms in ASD^64^. Moreover, it was previously reported that using *B. longum* subsp and *L. plantarum* WCFS1 was associated with alleviation of GI symptoms and immunomodulation of cytokines^65–67^.

The same observation in *Lactobacillus* genus and species was noted in accordance with the relative abundance of *B. longum* which was higher in the TD group, even though no difference at the genus level was reported. A higher level of this species in the TD group corresponds well with their numerous profound health benefits in humans as they play a role in the protection of the gut barrier, improving bowel regularity, alleviating symptoms of irritable bowel syndrome, suppressing oxidative stress, regulating pro-inflammatory and anti-inflammatory cytokines^68,69^.

The gut microbiome at the age of one year old can predict cognitive performance at the age of two years old, specifically in communication behaviours, indicating a potential link between the language developments or delayed cognitive and gut microbiome^70^. Age can affect the count and diversity of *Lactobacillus* species in the gut microbiota. During early stages of life, *Lactobacillus* species are predominated in the gut, especially of breastfed infant, and their relative abundance is often lowered during adulthood, however, individual variations may exist between different age groups. In contrast, Ghosh et al. reported the abundance of *Lactobacillus* in the elderly than in child/teen/young/middle-aged groups in most of the surveyed countries^71^. Similarly, in the current study, non-significant *Lactobacillus* count was positively correlated with the age of ASD children (r_s_=0.017). *L. plantarum* count was significantly increased with the increasing age of ASD children (r_s_=0.285, *p* = 0.007), which was observed in a previous study, where there was no decrease in the count of *L. plantarum* in ASD children aged from 8 to 10 years than those aged from 4 to 7 years^27^. Meanwhile, the abundance and diversity of *Bifidobacterium* species vary across different life stages and tend to decline gradually as individuals transition from infancy to adulthood. This finding was observed in the current study, where counts of the *Bifidobacterium* genus were significantly inversely proportional to the age of ASD children (r_s_= -0.228, *p* = 0.034)^72^.

Sex should thoroughly be considered in studies of ASD. In the current study, the majority of ASD children were males (66.7%), which aligns with the consistent male predominance described across several epidemiological studies^73–75^. This significantly higher prevalence in males has promoted various causal hypotheses regarding ASD, including theories of testosterone exposure in utero and increased vulnerability to early environmental influences that promote social competency^76–78^.

The complexity and diversity of gut microbiota can be greatly influenced by sex as *Firmicutes*, *Bacteroides* and *Lactobacillus* were reported to be higher among males^79^. In the present study, the predominance was for *Bifidobacterium* in TD males, and *Bifidobacterium* and *L. plantarum* in ASD females. This may be attributed to some factors such as maternal microbiome, mode of delivery, type of feeding and food selection^80^.

One of the limitations of the study at hand was the small sample size which can probably lead to the loss of statistical significance at certain points. Another limitation was the limited number of bacterial species.

## Conclusion

The current study highlighted alterations in psychobiotic bacteria in the gut of autistic children in comparison to typically developing children. Clearly, the impact of these alterations may play a role in the severity and sensory impairment of ASD cases. It has been observed that the psychobiotics were correlated with specific features of ASD, more than other types of bacteria. Hence, it is recommended to assess the gut microbiome profile before considering the use of these psychobiotics. Species-level analysis of an individual’s gut microbiome profile and clinical symptoms should be assessed prior to blind administration of probiotics to ASD cases.

## Electronic supplementary material

Below is the link to the electronic supplementary material.

Supplementary Material 1

## Data Availability

The datasets used and analysed during the current study are available from the corresponding author upon reasonable request.

## Abbreviations

ASD: Autism Spectrum Disorder
ATEC: Autism Treatment Evaluation Checklist
CARS: Childhood Autism Rating Scale
DSM-5: Diagnostic and Statistical Manual of Mental Disorders Fifth Edition criteria
DNA: Deoxyribonucleic acid
GI: Gastrointestinal
6-GSI: Modified short version of the GI Severity Index
GABA: γ-Aminobutyric acid
H: Kruskal Wallis test
IL-10: Interleukin-10
IL-12: Interleukin-12
IQR: Inter quartile range
IBM SPSS software: Statistical package for the social sciences
PCR analysis: Poly Chain Reaction analysis
rs: Spearman’s correlation
RNA: Ribonucleic acid
SD: Standard deviation
SRS: Social responsiveness scale
SSP: Short Sensory Profile
TD: typically developing children
T-test: Hypothesis test statistic
U: Mann Whitney test
16S rRNA: 16 svedberg ribosomal ribonucleic acid
16S rDNA: 16 svedberg ribosomal deoxyribonucleic acid
