# Supplementary material for: Unraveling the relative abundance of psychobiotic bacteria in children with Autism Spectrum Disorder
Source: Sci Rep. 2024 Oct 17;14:24321. doi: 10.1038/s41598-024-72962-3 (PMC11484847; doi:10.1038/s41598-024-72962-3)
Supplement: Supplementary file 1 — Supplementary Material 1 [file 41598_2024_72962_MOESM1_ESM.docx]

**Supplementary tables:**

**Table S1** Primers used in the current study

| **Target** | **Primer Name** | **Primer Sequence (5'-3')** | **References** |
| --- | --- | --- | --- |
| **Total bacteria** | UnivF  UnivR | TCCTACGGGAGGCAGCAGT  GGACTACCAGGGTATCTATCCTGTT | 28 |
| ***Bifidobacterium*** | Bif-F  Bif-R | TCGCGTC[C/T]GGTGTGAAAG  CCACATCCAGC[A/G]TCCAC | 27 |
| ***B. longum*** | *B. longum* F  *B. longum R* | CAG TTG ATC GCA TGG TCT T  TAC CCG TCG AAG CCA C | 27 |
| ***Lactobacillus*** | Lacto-F  Lacto-R | AGCAGTAGGGAATCTTCCA  CACCGCTACACATGGAG | 26 |
| ***L. plantarum*** | *L. plantarum F*  *L. plantarum R* | ATTCATAGTCTAGTTGGAGGT  CCTGAACTGAGAGAATTTGA | 26 |
| ***L. reuteri*** | *L. reuteri F*  *L. reuteri R* | GAAGATCAGTCGCAYTGGCCCAA  TCCATTGTGGCCGATCAG | 26 |

| **Table S2** Comparison between males and females according to the psychobiotic bacteria in TD group. (n= 36) | | | | | |
| --- | --- | --- | --- | --- | --- |
| **Bacteria** | **Group** | | **U** | ***p*-value** |  |
|  | **Male (n = 20)**  **Median (IQR)** | **Female (n = 16)**  **Median (IQR)** |  |  |  |
| *Lactobacillus* | 3.66E-3 (2.51E-2) | 3.03E-3 (5.18E-2) | 160.00 | 1.000 |  |
| *L. reuteri* | 7.99E-5 (3.60E-4) | 6.39E-5 (2.17E-4) | 134.50 | 0.422 |  |
| *L. plantarum* | 1.46E-5 (4.20E-5) | 1.64E-5 (8.17E-4) | 136.00 | 0.459 |  |
| *Bifidobacterium* | 1.25E-1 (2.40E-1) | 2.91E-2 (6.67E-2) | **84.00^*^** | **0.015^*^** |  |
| *B. longum* | 1.44E-2 (3.09E-2) | 5.51E-3 (1.75E-2) | 149.00 | 0.741 |  |
| **IQR:** Inter quartile range  **U:** Mann Whitney test.  ***p*-value:** *p* value for comparing between the two studied groups  *: Statistically significant at *p* ≤ 0.05 | | | | | |

| **Table S3** Comparison between males and females according to the psychobiotic bacteria in ASD children. (n= 87) | | | | | |
| --- | --- | --- | --- | --- | --- |
| **Bacteria** | **Group** | | **U** | ***p*-value** |  |
|  | **Male (n = 58)**  **Median (IQR)** | **Female (n = 29)**  **Median (IQR)** |  |  |  |
| *Lactobacillus* | 3.79E-3 (1.05E-2) | 3.10E-3 (1.46E-2) | 789.00 | 0.640 |  |
| *L. reuteri* | 2.14E-5 (5.59E-5) | 1.30E-5 (3.46E-5) | 692.00 | 0.180 |  |
| *L. plantarum* | 6.70E-6 (3.16E-5) | 1.04E-5 (2.16E-5) | 793.00 | 0.665 |  |
| *Bifidobacterium* | 4.22E-2 (1.04E-1) | 7.91E-2 (1.28E-1) | 646.00 | 0.079 |  |
| *B. longum* | 7.45E-3 (1.52E-2) | 5.41E-3 (3.35E-2) | 816.00 | 0.822 |  |
| **IQR:** Inter quartile range  **U:** Mann Whitney test.  ***p*-value:** *p* value for comparing between the two studied groups  *: Statistically significant at *p* ≤ 0.05 | | | | | |

| **Table S4** Clinical characteristics of the ASD cases. | | | | |
| --- | --- | --- | --- | --- |
| **Metric** | **CARS** | **Total ATEC** | **Total SSP** | **Total GIS** |
| **Mean** | 33.64 | 79.26 | 127.94 | 3.37 |
| **SD** | 5.14 | 24.35 | 16.07 | 2.23 |
| **Median** | 33.00 | 80.00 | 131.00 | 3.00 |
| **Range** | 34.00 | 77.00 | 98.00 | 10.00 |
| **IQR** | 5.00 | 40.00 | 20.50 | 3.00 |
| **IQR :** Inter quartile range **SD:** Standard deviation | | | | |

**Table S****5** Descriptive analysis of ASD children according to ATEC subscale

| **ATEC subscales** | **Min. – Max.** | **Mean ± SD.** | **Median (IQR)** |
| --- | --- | --- | --- |
| **Speech/Language/Communication** | 0.0 – 28.0 | 18.26 ± 6.56 | 20.0 (16.0 – 22.50) |
| **Sociability** | 4.0 – 31.0 | 18.83 ± 6.97 | 19.0 (15.0 – 23.0) |
| **Sensory/Cognitive Awareness** | 6.0 – 30.0 | 18.26 ± 6.34 | 18.0 (14.50 – 23.50) |
| **Health/Physical/Behavior** | 6.0 – 54.0 | 24.29 ± 13.21 | 21.0 (15.0 – 33.0) |
| **Total ATEC** | 41.0 – 118.0 | 79.63 ± 24.88 | 80.0 (59.0 – 99.0) |

**IQR:** Inter quartile range **SD:** Standard deviation

**Table S6** Distribution of the studied cases according to SSP subscales in ASD (n = 87)

| **SSP subscales** | **No.** | **%** |
| --- | --- | --- |
| **Tactile** |  |  |
| Definite difference (7 – 26) | 59 | 67.8 |
| Probable difference (27 – 29) | 12 | 13.8 |
| Typical performance (30 – 35) | 16 | 18.4 |
| Min. – Max. | 11.0 – 35.0 | |
| Mean ± SD. | 24.20 ± 5.39 | |
| Median (IQR) | 23.0 (20.0 – 27.5) | |
| **Taste/smell** |  |  |
| Definite difference (4 – 11) | 31 | 35.6 |
| Probable difference (12 – 14) | 21 | 24.1 |
| Typical performance (15 – 20) | 35 | 40.2 |
| Min. – Max. | 4.0 – 20.0 | |
| Mean ± SD. | 13.20 ± 4.42 | |
| Median (IQR) | 13.0 (9.50 – 17.0) | |
| **Movement** |  |  |
| Definite difference (3 – 10) | 17 | 19.5 |
| Probable difference (11 – 12) | 20 | 23.0 |
| Typical performance (13 – 15) | 50 | 57.5 |
| Min. – Max. | 3.0 – 15.0 | |
| Mean ± SD. | 12.64 ± 3.13 | |
| Median (IQR) | 15.0 (11.0 – 15.0) | |
| **Under responsive** |  |  |
| Definite difference (7 – 23) | 79 | 90.8 |
| Probable difference (24 – 26) | 6 | 6.9 |
| Typical performance (27 – 35) | 2 | 2.3 |
| Min. – Max. | 7.0 – 31.0 | |
| Mean ± SD. | 16.93 ± 5.04 | |
| Median (IQR) | 16.0 (13.0 – 20.50) | |
| **Auditory** |  |  |
| Definite difference (6 – 19) | 57 | 65.5 |
| Probable difference (20 – 22) | 17 | 19.5 |
| Typical performance (23 – 30) | 13 | 14.9 |
| Min. – Max. | 6.0 – 28.0 | |
| Mean ± SD. | 17.66 ± 4.53 | |
| Median (IQR) | 18.0 (14.0 – 20.50) | |
| **Low Energy** |  |  |
| Definite difference (6 – 23) | 32 | 36.8 |
| Probable difference (24 – 25) | 11 | 12.6 |
| Typical performance (26 – 30) | 44 | 50.6 |
| Min. – Max. | 6.0 – 30.0 | |
| Mean ± SD. | 24.34 ± 6.38 | |
| Median (IQR) | 26.0 (20.50 – 30.0) | |
| **Visual** |  |  |
| Definite difference (5 – 15) | 16 | 18.4 |
| Probable difference (16 – 18) | 17 | 19.5 |
| Typical performance (19 – 25) | 54 | 62.1 |
| Min. – Max. | 5.0 – 25.0 | |
| Mean ± SD. | 18.98 ± 4.80 | |
| Median (IQR) | 20.0 (17.0 – 22.0) | |

IQR: **Inter quartile range** SD: **Standard deviation**

**Table S7** Relation between 6-GSI total score and bacteria in ASD children

| **Bacteria** | **6-GSI Total score** | | | **H** | ***p*** | **r_s_** | ***p*** |
| --- | --- | --- | --- | --- | --- | --- | --- |
|  | **No GIT Symptoms (0) (n = 10)** | **Moderate (1 – 3) (n = 35)** | **Severe (≥4) (n = 42)** |  |  |  |  |
| ***Lactobacilli*** |  |  |  |  |  | 0.031 | 0.778 |
| Min. – Max. | 9.31E-5 – 7.43E-2 | 1.68E-5 – 3.94E-1 | 5.12E-5 – 2.50E-1 | 1.835 | 0.400 |  |  |
| Mean ± SD. | 1.28E-2 ± 2.36E-2 | 4.26E-2 ± 9.15E-2 | 2.58E-2 ± 5.39E-2 |  |  |  |  |
| Median | **3.97E-4** | **3.91E-3** | **3.38E-3** |  |  |  |  |
| IQR | 2.77E-4 – 1.66E-2 | 1.40E-3 – 1.38E-2 | 6.32E-4 – 1.05E-2 |  |  |  |  |
| ***L. reuteri*** |  |  |  |  |  | -0.001 | 0.989 |
| Min. – Max. | 1.76E-6 – 1.90E-3 | 0.0E+0 – 7.44E-3 | 0.0E+0 – 7.27E-2 | 0.022 | 0.989 |  |  |
| Mean ± SD. | 2.08E-4 ± 5.95E-4 | 6.42E-4 ± 1.74E-3 | 1.81E-3 ± 1.12E-2 |  |  |  |  |
| Median | **1.52E-5** | **1.50E-5** | **1.69E-5** |  |  |  |  |
| IQR | 3.25E-6 – 4.04E-5 | 2.89E-6 – 9.94E-5 | 4.14E-6 – 4.21E-5 |  |  |  |  |
| ***L. plantarum*** |  |  |  |  |  | -0.039 | 0.720 |
| Min. – Max. | 0.0E+0 – 1.29E-4 | 0.0E+0 – 8.48E-2 | 0.0E+0 – 1.91E-4 | 0.508 | 0.776 |  |  |
| Mean ± SD. | 1.87E-5 ± 3.92E-5 | 2.78E-3 ± 1.44E-2 | 2.60E-5 ± 4.89E-5 |  |  |  |  |
| Median | **6.70E-6** | **1.02E-5** | **5.42E-6** |  |  |  |  |
| IQR | 1.92E-6 – 1.04E-5 | 1.24E-6 – 3.96E-5 | 1.56E-6 – 2.85E-5 |  |  |  |  |
| ***Bifidobacteria*** |  |  |  |  |  | 0.099 | 0.359 |
| Min. – Max. | 2.07E-3 – 1.38E-1 | 1.05E-3 – 3.46E-1 | 1.27E-3 – 3.97E-1 | 4.313 | 0.116 |  |  |
| Mean ± SD. | 3.14E-2 ± 3.94E-2 | 9.79E-2 ± 1.05E-1 | 9.29E-2 ± 9.94E-2 |  |  |  |  |
| Median | **2.13E-2** | **7.03E-2** | **5.67E-2** |  |  |  |  |
| IQR | 9.76E-3 – 3.06E-2 | 9.98E-3 – 1.28E-1 | 2.48E-2 – 1.28E-1 |  |  |  |  |
| ***B. longum*** |  |  |  |  |  | 0.086 | 0.429 |
| Min. – Max. | 1.91E-4 – 1.27E-2 | 2.20E-4 – 2.92E-1 | 4.72E-5 – 1.92E-1 | **6.405^*^** | **0.041^*^** |  |  |
| Mean ± SD. | 3.84E-3 ± 4.23E-3 | 2.83E-2 ± 5.37E-2 | 2.15E-2 ± 3.87E-2 |  |  |  |  |
| Median | **2.58E-3** | **8.49E-3** | **6.89E-3** |  |  |  |  |
| IQR | 7.69E-4 – 4.65E-3 | 4.05E-3 – 3.32E-2 | 2.06E-3 – 1.80E-2 |  |  |  |  |
| **Significance between Subgroups.** | *p_1_*=0.011^*^, *p_2_*=0.038^*^, *p_3_*=0.437 | | |  |  |  |  |

IQR: Inter quartile range SD: Standard deviation

H: H for Kruskal Wallis test, Pairwise comparison bet. each 2 groups was done using Post Hoc Test (Dunn's for multiple comparisons test)

*p*: *p* value for comparing between the three sub groups of GSI Total Score

*p*_1_: *p* value for comparing between Normal (0) and Moderate (1 – 3)

*p*_2_: *p* value for comparing between Normal (0) and Severe (≥4)

*p*_3_: *p* value for comparing between Moderate (1 – 3) and Severe (≥4)

*: Statistically significant at p ≤ 0.05

**Supplementary figures:**

**
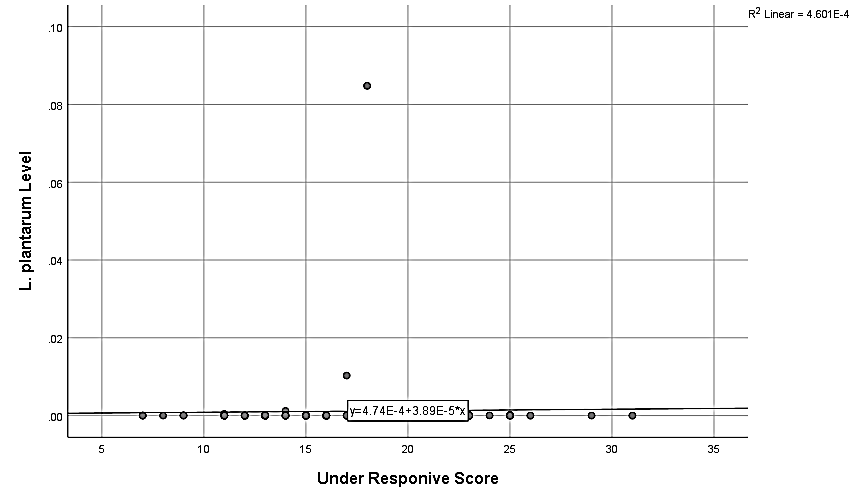
**

**Figure S1:** The correlation between under-responsive scores and *L.* *plantarum* levels.


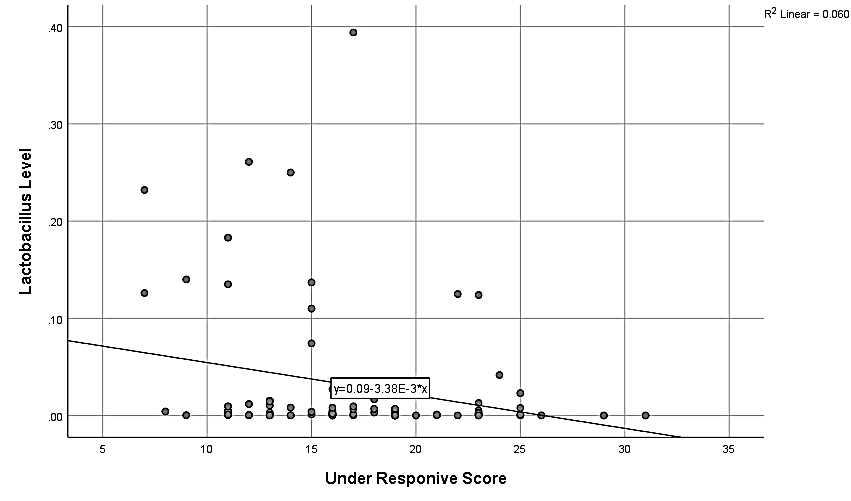


**Figure S2:** The correlation between under-responsive scores and *Lactobacillus* levels.
